# microRNA-194 is increased in polycystic ovary syndrome granulosa cell and induce KGN cells apoptosis by direct targeting heparin-binding EGF-like growth factor

**DOI:** 10.1186/s12958-021-00850-w

**Published:** 2021-11-23

**Authors:** Yi-xuan Wu, Yan-shan Lin, Si-chen Li, Xi Yao, Mingwei Cheng, Lin Zhu, Hai-ying Liu

**Affiliations:** 1 Department of Obstetrics and Gynecology, Center for Reproductive Medicine, Key Laboratory for Major Obstetric Diseases of Guangdong Province, The Third Affiliated Hospital of Guangzhou Medical University, Guangzhou, China; 2 Present Address: Key Laboratory for Reproductive Medicine of Guangdong Province, The Third Affiliated Hospital of Guangzhou Medical University, 63 Duobao Road, Guangdong, Guangzhou, China

**Keywords:** miRNA, Cell growth, Granulosa cells, PCOS

## Abstract

**Background:**

Polycystic ovary syndrome (PCOS) is an endocrine-related follicular developmental disorder that affects 50 %-70 % of reproductive-aged women diagnosed with ovulation-related infertility. Abnormal proliferation and apoptosis of granulosa cells (GCs) are thought to be the critical factors leading to abnormal maturation of follicles. It has been shown that microRNAs (miRNAs) exert a significant influence in the pathogenesis of PCOS; however, the relationship between miRNA, PCOS, and GC apoptosis is not entirely understood.

**Methods:**

To clarify the effect of miR-194 in PCOS, CCK-8, Ki67 staining, AO/EB, and flow cytometry assays were used to assess cell growth, proliferation, and apoptosis in KGN cells, which were artificially stimulated to overexpress miR-194. Luciferase reporter assays and rescue experiments were used to elucidate the mechanism underlying miR-194 in PCOS.

**Results:**

miR-194 expression was significantly up-regulated in rat models of PCOS and the ovarian GCs of PCOS patients. miR-194 suppression promoted KGN cell growth and proliferation. miR-194 overexpression also induced cell apoptosis, while miR-194 downregulation had an opposite effect. Furthermore, up-regulating heparin-binding EGF-like growth factor (HB-EGF) expression rescued the pro-apoptotic effects of miR-194 upregulation on KGN cells.

**Conclusions:**

miR-194 is increased in PCOS granulosa cell and may function as a novel biomarker and therapeutic target for KGN cells via HB-EGF regulation.

**Supplementary Information:**

The online version contains supplementary material available at 10.1186/s12958-021-00850-w.

## Background

Polycystic ovary syndrome (PCOS) is an endocrine disorder that affects female fertility. It has been reported that 5 %-10 % of women are infertile because of PCOS [1, 2], which is characterized by metabolic disorders, such as insulin resistance, infertility, ovulation failure, hyperandrogenism, and menstrual disorders, which are detrimental to a patient’s overall mental and physiologic well-being [3]. Studies have demonstrated that FSH promotes granulosa cell (GC) proliferation and differentiation [4]. Follicular atresia is a significant cause of female infertility. Indeed, this phenomenon is closely related to ovarian follicle development and GC apoptosis, and GC apoptosis is thought to be the primary mechanism underlying follicular atresia. Given the high prevalence of PCOS, the work had the purpose of identifying a new biomarker and therapeutic target for PCOS.

MicroRNAs (miRNAs) are ~22nt long small and non-coding RNAs that directly target the 3′-UTR region of mRNAs, thus affecting mRNA transcription and translation [5, 6]. miRNAs have been reported to have essential functions in metabolic disorders, including PCOS [7–9]. Specifically, miR-206 promotes LPS-induced inflammation and release of amyloid-beta by targeting IGF1 in microglia [10]. The miR-200 family (miR-200b and miR-200c) is up-regulated in GCs and inhibits KGN cell growth by repressing PTEN [11]. Additionally, miR-874-3p induces GC apoptosis by suppressing HDAC1-mediated p53 deacetylation due to testosterone exposure [12]. Furthermore, miR-103 has likely been associated with PCOS through its effect on IRS1 and subsequent modulation of the PI3K/AKT signaling pathway [13]. These findings showed that miRNAs are well-established in post-transcriptional regulation involved in PCOS-related molecular expression and GC apoptosis in PCOS.

In the current study, we focused on miR-194 because previous studies have alluded to its tumor-suppressive effects. Wang et al. [14]. reported that miR-194 expression is downregulated in gastric cancer tissues. Sun et al. [15]. showed that miR‐194 suppresses SSH2 expression in colon cancer stem cells. Li et al. [16] suggested that miR-194 functions inhibit laryngeal squamous cell carcinoma via Wee1 suppression. In addition, in prostate cancer, miR-194 can inhibit human nuclear distribution protein expression leading to an overall antitumor effect [17]. Despite the wealth of evidence regarding various functions of miR-194, little is known regarding the impact of miR-194 on GCs in PCOS.

Heparin-binding EGF-like growth factor (HB-EGF) was first detected in the brainstem and shown to play a role in neuronal and glial maturation. HB-EGF protects against phenotypes related to metabolic syndrome and advanced metabolic diseases, suggesting that HB-EGF is a potential target in metabolic disorders [18]. HB-EGF has been reported to be related to cell proliferation, migration, and inflammation in many diseases [19]. In addition, HB-EGF has been shown to have an essential role in ovarian maturation. Shen et al. [20] suggested that HB-EGF regulates the development of ovarian cancer cells. Robertson et al. [21]. reported that HB-EGF may be involved in embryo loss and fetal programming regulated by cytokines that are toxic to the growing embryo; however, the effects of HB-EGF on GC cells in PCOS have not been established.

Therefore, the purpose of our experiments is to explore the expression of miR-194/HB-EGF in GCs of PCOS patients and a PCOS rat model. Also, investigate the role and effect of miR-194/HB-EGF in KGN cell growth, proliferation, and apoptosis to the underlying mechanism of miR-194/ HB-EGF in PCOS progress.

## Materials and methods

### Experimental animals

Sixteen 21-day-old female SD rats were purchased from the Beijing Vital River Laboratory Animal Technology Co., Ltd. (Beijing, China). Animal protocols were approved by the Animal Care and Use Committee of Guangzhou Medical University (protocol number: NX-2019-023). Rats were randomly divided into a control cohort (n = 8) or the PCOS cohort (n = 8). All animals were maintained at room temperature (22–26 °C) and a 12-h light/dark cycle with filtered air and access to food and water *ad libitum*. Briefly, eight female rats received subcutaneous injections of Dehydroepiandrosterone (DHEA) daily (6 mg/100 g body weight [100 µl/rat in sesame oil with 10 % 95 % ethanol]; Sigma, daily for 20 consecutive days. All animals were sacrificed via cervical vertebrae dislocation (CVD) upon completion of the experiments. Hematoxylin and eosin (H&E) staining of rat ovaries and ELISA kit of serum results showed that the PCOS rat model was established (Fig. S1). Serum levels of FSH, LH, E2, and T were quantified to confirm the validity of the PCOS model.

### Patient samples

Twenty females (<35 years of age) who were undergoing planned *in vitro* fertilization (IVF) at our fertility center between December 2019 and November 2020 were enrolled in the current study and selected for further analysis of their ovarian GCs. Ten of the patients were diagnosed with PCOS based on the Rotterdam criteria (Rui Wang et al. 2017). Ten patients had regular menstrual cycles (non-PCOS group) and male infertility. None of the patients were treated with exogenous hormones for at least six months prior to enrollment in this study. Women with endometriosis and a history of recurrent abortions, genetic diseases, low ovarian function, and other systemic chronic diseases were excluded. All participants provided informed consent prior to inclusion. The Institutional Ethics Committee approved all study protocols of the Third Affiliated Hospital of Guangzhou Medical University (Ethics Review Board, 2018NO:083; Guangzhou, China).

### Granulosa cells

Follicular fluid (FF) from 20 patients was collected from a pooled follicular aspirate during oocyte retrieval. The FF was centrifuged at four °C (Thermo Fisher Scientific, Shanghai, China), washed with 4 ml of lymphocyte separation medium (Biosharp, Shanghai, China), lysed with 2 ml of red blood cell lysis solution (Biosharp), digested with 200 ul of 0.25 % EDTA-trypsin (Gibco, Grand Island, NY, USA), and creened with 70 mm to obtain GCs. All GC samples were stored at -80 °C for protein and RNA analyses.

### Cell culture and treatment

KGN cells were purchased from the Riken Cell Bank (Wako, Saitama, Japan) [22]. KGN cells were cultured in high-glucose conditions using DMEM (Gibco) containing 10 % FBS (Gibco), 100 U/ml penicillin, and 100 µg/ml of streptomycin. All cells were maintained at 5 % CO_2_ with 37 °C. Transfection with a blank vector and a miR-194 mimic or inhibitor (RiboBIO, Guangzhou, China) was then carried out using RiboFECT^TM^ CP (RiboBIO).

### CCK-8 assay

The CCK-8 assay kit (Dojindo Molecular Technologies, Japan) was used to assess cell growth. KGN cells (2 × 10^3^) were plated for 24 h in 96-well plates before treatment (miR-194 mimic, miR-194 inhibitor, or miR-194 mimic+HB-EGF) for 48 h. At the end of the treatment period, all cells were exposed for two h to 10 µl of CCK-8 reagent in a 5 % CO_2_ atmosphere at 37 °C. A microplate reader was then used to visualize cell growth at 450 nm (Thermo Scientific).

### AO/EB assay

An AO/EB assay kit (Solarbio, Beijing, China) was used to detect cell apoptosis. KGN cells (1 × 10^3^) were first transfected with miR-194 mimic, miR-194 inhibitor, or miR-194 mimic+HB-EGF for 48 h, were exposed to 20 µM AO/EB (1:1) in 1 ml of PBS for 2 - 5 min, followed by a PBS rinse every 15 min. A fluorescence microscope (PH-YGD; Phoenix Co., ShangRao, China) was utilized to detect apoptosis-positive cells.

### Flow cytometry

The cell apoptosis rate was detected using a relevant kit (Wanleibio, Shenyang, China). Briefly, KGN (1 × 10^4^) cells were transfected for 48 h with a miR-194 mimic, miR-194 inhibitor, or miR-194 mimic+HB-EGF, then processed using trypsin. FITC (5 µL) and 10 µL of PI were then added. A flow cytometer (Phoenix Co.) was used to detect the apoptotic rate.

### qRT-PCR

RNAs were extracted using TRIzol reagent (Thermo Fisher Scientific, Sunnyvale, CA, USA). cDNA was synthesized using a SuperScript II first-strand cDNA synthesis kit (Thermo Fisher Scientific). A TaqMan MicroRNA assay (Thermo Fisher Scientific) was used to facilitate the qRT-PCR reaction on an ABI Prism 7900 detection system. Relative gene expression was evaluated using the 2 − ΔΔCq method. Table 1 shows the relevant PCR primers used in this study.

**Table 1:** Primers for qRT-PCR

5’-3' 3’-5'	
HBEGF: CGGGGAGTGCAGATACCTG, TTCTCCACTGGTAGAGTCAGC;	
GAPDH: GTCTCCTCTGACTTCAACAGCG, ACCACCCTGTTGCTGTAGCCAA;	
miR-194: ATGGACCTGGGGCCACGAAG, TCTGGCCTGGGAGCGTCG	
U6 TTCGTGAAGCGTTCCATATTTT	

### Western blot

Total protein was extracted from KGN cells and ovarian tissues. Samples (60 – 80 mg of protein) were subsequently separated with 10 %~12.5 % SDS-PAGE gels for two h using a 300-mA electric current. Non-fat milk was used to treat membranes for 2 h at 4 °C before probing overnight with antibodies against HB-EGF (catalog # 15,071; Cell Signaling Technology, USA), p53 (catalog # wL01919; Wanleigio), p21 (catalog # WLH0362; Wanleigio), p16 (catalog # WLH3673; Wanleigio) and β-actin (catalog # 3700; Cell Signaling Technology). After that, membranes were incubated with infrared (IR) fluorescent dye-conjugated secondary antibodies (1:10,000 – 20,000 dilution; LI-COR Biosciences, Lincoln, NE, USA) for 1 h. An LI-COR Biosciences Infrared Imaging System was used to detect blots with the band intensity quantified using LI-COR Biosciences Odyssey 3.0 software. All values were standardized against β-actin expression.

### Luciferase reporter assay

The Mut sequences or WT binding sites of the HB-EGF 3′-UTR were transfected into the pGL3 promoter vector SacI and KpnI sites (RiboBIO). The RiboFECT^TM^ CP system (RiboBIO) was used to transfect KGN cells with pGL3-HB-EGF-WT or pGL3-HB-EGF-MUT, as well as miR-194 mimics or miR-NC mimic (RiboBIO). A luciferase reporter assay (Promega, Madison, WI, USA) was used to evaluate luciferase activity.

### miRNA-target gene interactions

Potential miR-194 gene targets were predicted using four databases, including Targetscan [23], PicTar5 [24], and PITA [25] databases. Table 2 depicts a total of 29 miR-194 target genes that were identified.

**Table 2:** The genes intersection of bioinformatics websites

Common elements in PITA & TargetaScan & PicTar and miRD:	
ARHGAP21 CSNK1D RSBN1L NRN1 STX16 MID1IP1 WAPAL TSPAN7 NAA50 HOOK3 MEIS2 EPC2 FOXA1 SPCS2 SETD8 ITPKB HNF1B PTPN12 UBR3 LRRFIP1 HB-EGF SEPHS1 GYG1 TLN2 C7orf60 BCKDHA LUC7L3 DUSP9 SSH2	

### ELISA assay

ELISA kits were used to assess serum levels of FSH, LH, and T and E2 (catalog #’s H101-1-2, H206-1-2, and H102-1, respectively; Nanjing Jiancheng Bioengineering Institute, Nanjing, Jiangsu, China).

### TUNEL and ki67 assay

KGN cells were first exposed to miR-194 mimic, miR-NC mimic, or miR-194 mimic+HBEGF for 48 h before processing for 30 min with 4 % formaldehyde. Cells were subsequently processed according to protocols provided by WanLeiBIO. An apoptosis detection kit (catalog # WLA030; WanLeiBIO) enabled us to perform the TUNEL assay as previously described [26, 27]. The sections were photographed under a fluorescent microscope within 2 h. Image J software was used to quantify the cell apoptosis rate.

Ki67 expression was determined as previously described. Image J software was used to quantify Ki67 expression after treatment with miR-194 or miR-NC inhibitor in KGN cells.

### Statistical analysis

All data are depicted as the mean ± SEM and analyzed using SPSS version 21.0 software (IBM Corp., Armonk, NY, USA). Student’s t-test was used to analyze variances between two groups, while multi-group comparisons were assessed using analysis of variance (ANOVA). The level of significance was set as P < 0.05.

## Results

### miR-194 expression was up-regulated in PCOS GCs

**Fig. 1:**
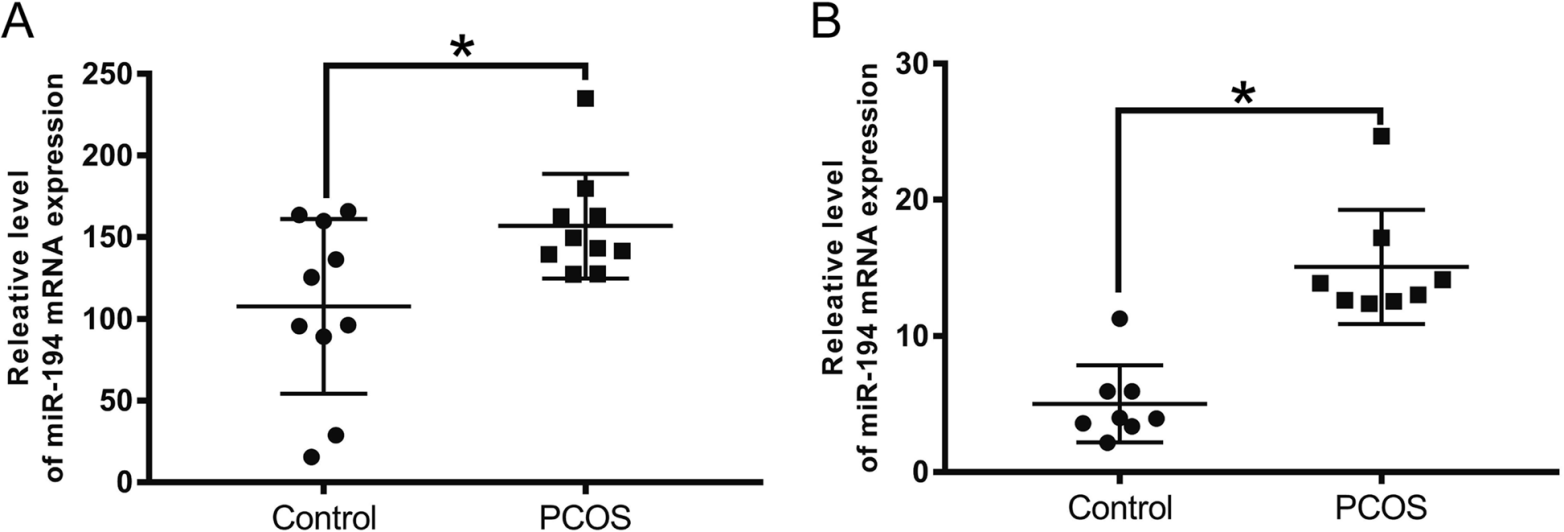
PCOS GCs harbored up-regulated miR-194 expressions. qRT-PCR was used to evaluate miR-194 expressions in PCOS GCs (**A**) and PCOS rat ovarian tissues (**B**). 𝑛 = 3-6 for each group. *P < 0.05 vs. control group

miR-194 expression was up-regulated in the GCs of PCOS patients in contrast to the normal group as shown by qRT-PCR (Fig. 1 A).

### miR-194 expression was increased in the PCOS rat model

We determined the effect of miR-194 *in vivo*. As shown in Figure S1, DHEA-treated rats presented PCOS phenotypes, including the increasing weight of the ovary, polycystic, etc. The changed serum hormone levels (Follicle-stimulating hormone(FSH), Estradiol(E2), Testosterone(T), and luteinizing hormone(LH)) also testified the pathogenesis of PCOS rats (Figure S1). The ovarian miR-194 expression was increased in the rat model compared to control rats (Fig. 1B). This finding serves to further highlight the role of miR-194 as a potential PCOS promoter.

### miR-194 suppressed KGN cell growth while inducing cell apoptosis

**Fig. 2:**
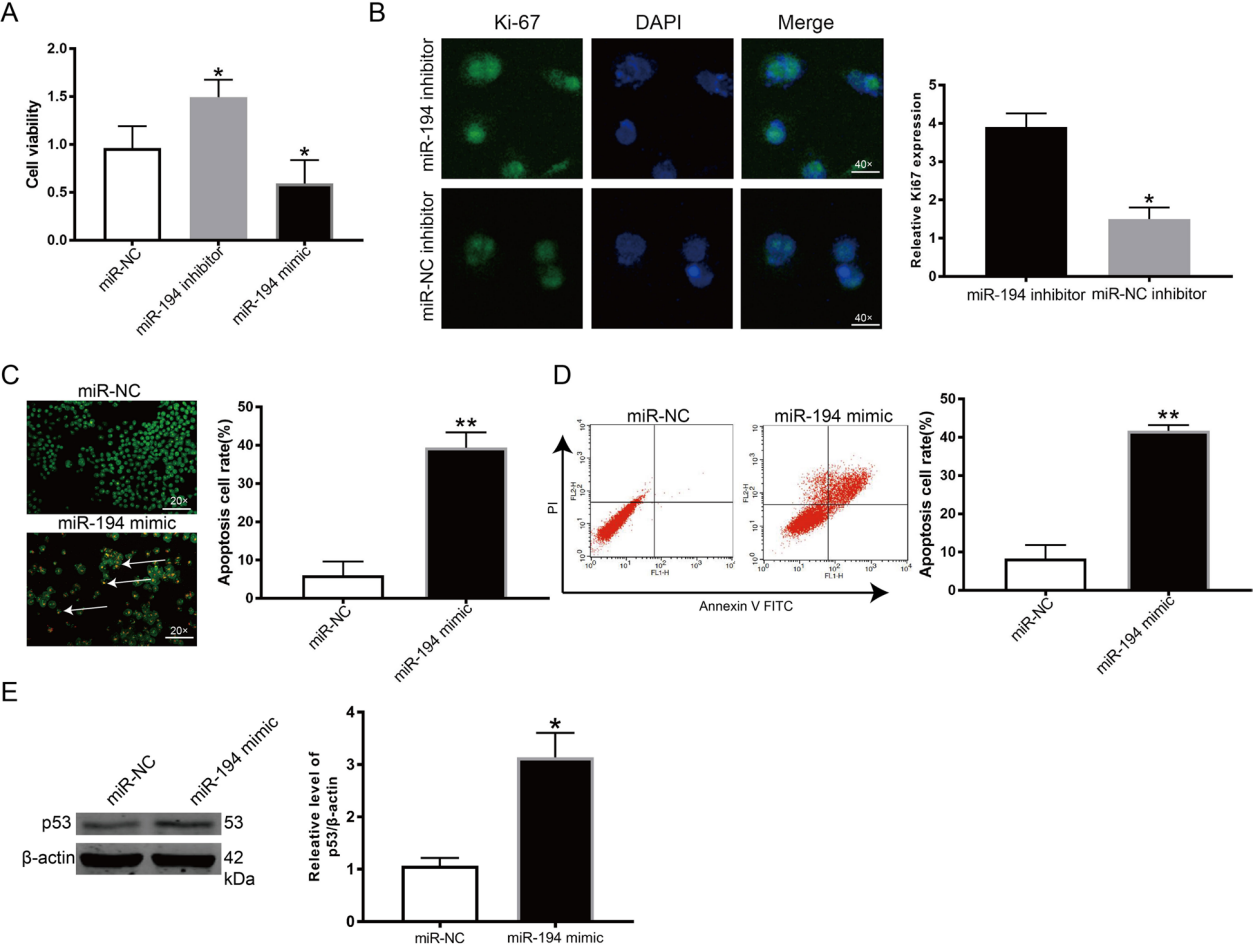
miR-194 regulated KGN cell growth and apoptosis. (**A**) A CCK-8 assay assessed KGN cell viability upon treatment with miR-194 mimic or inhibitor. (**B**) Ki67 was utilized to determine the proliferative ability of KGN cells upon transfection with miR-194 or miR-NC inhibitors. (**C**-**D**) The AO/EB and flow cytometry were used to detect the KGN cell apoptosis. (**E**) p53, p21 and p16 protein level after treatment with miR-194 or miR-NC mimic. 𝑛 = 3-6 for each group. *P < 0.05 vs. miR-NC group

We then hypothesized that miR-194 functions to promote PCOS progression, given the aberrant miR-194 expression in KGN cells. To confirm our hypothesis, we artificially knocked down or stimulated miR-194 expression in KGN cells with a miR-194 inhibitor or mimic, respectively. KGN cell viability was significantly downregulated in cells exposed to miR-194 mimic but was up-regulated in cells exposed to the miR-194 inhibitor (Fig. 2 A). The impact of miR-194 on cell proliferation was investigated using the Ki67 assay. miR-194 inhibition promoted KGN cell proliferation compared with the control group (Fig. 2B). Both AO/EB and flow cytometry were used to demonstrate that KGN cell apoptosis was increased by miR-194 upregulation. Increased miR-194 expression induced KGN cell apoptosis compared with the control group (Fig. 2 C, D). p53,p21, and p16 protein expression were increased after transfection with a miR-194 mimic in KGN cells (Fig. 2E).

#### miR-194 directly targeted HB-EGF

**Fig. 3:**
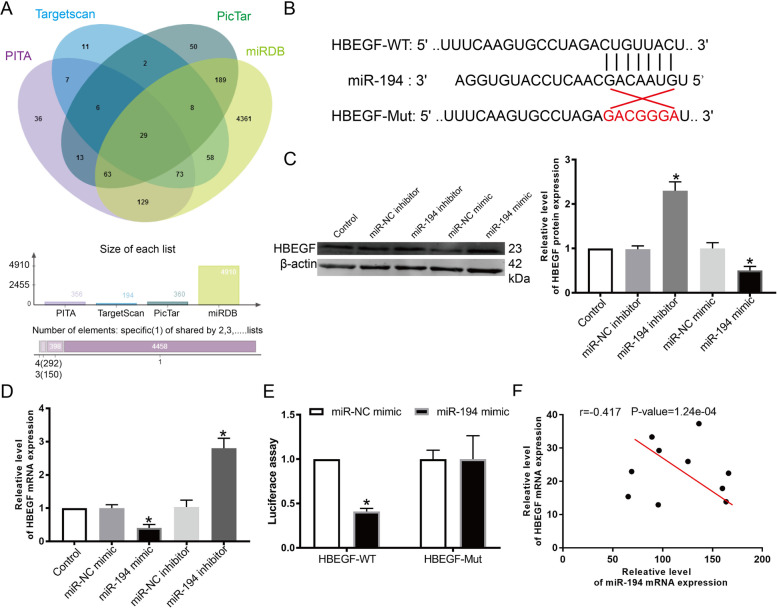
HB-EGF was directly targeted by miR-194. ** A** Venn diagram demonstrates the intersection of data extracted from several bioinformatics websites. (**B**) Binding sites shared between miR-194 and HB-EGF. (**C**-**D**) HB-EGF protein and mRNA expression in the GCs. KGN cells were transfected for 48 h with miR-194 mimic, miR-194 inhibitor, or the miR-NC group. (**E**) Luciferase reporter assays. (**F**) miR-194 and HB-EGF mRNA expression in PCOS patient ovarian GCs. n= 3-6 for each group; *P < 0.05 vs. control group or miR-NC group

miRNAs are known post-transcriptional modifiers and directly bind to the 3’UTR region of their target genes(Li et al. [28]). Bioinformatics methods enabled the prediction of the miR-194 target gene; specifically, HB-EGF was shown to be a potential target gene (Fig. 3 A and Table 2). We then determined the relationship between miR-194 and HB-EGF using WT-HBEGF and MUT-HBEGF binding units of miR-194, as shown in Fig. 3B. miR-194 mimics repressed HB-EGF protein and mRNA expression in KGN cells (Fig. 3 C, D). In addition, co-transfection of WT-HB-EGF with a miR-194 mimic decreased activity in the luciferase reporter assay. In contrast, co-transfection of MUT-HB-EGF with a miR-194 mimic yielded no significant change (Fig. 3E). We also showed that HB-EGF and miR-194 were negatively regulated in PCOS GCs (r=-0.417, P=1.24e-04, Fig. 3 F).

### HB-EGF expression was downregulated in PCOS GCs and ovarian tissues of a PCOS rat model

**Fig. 4:**
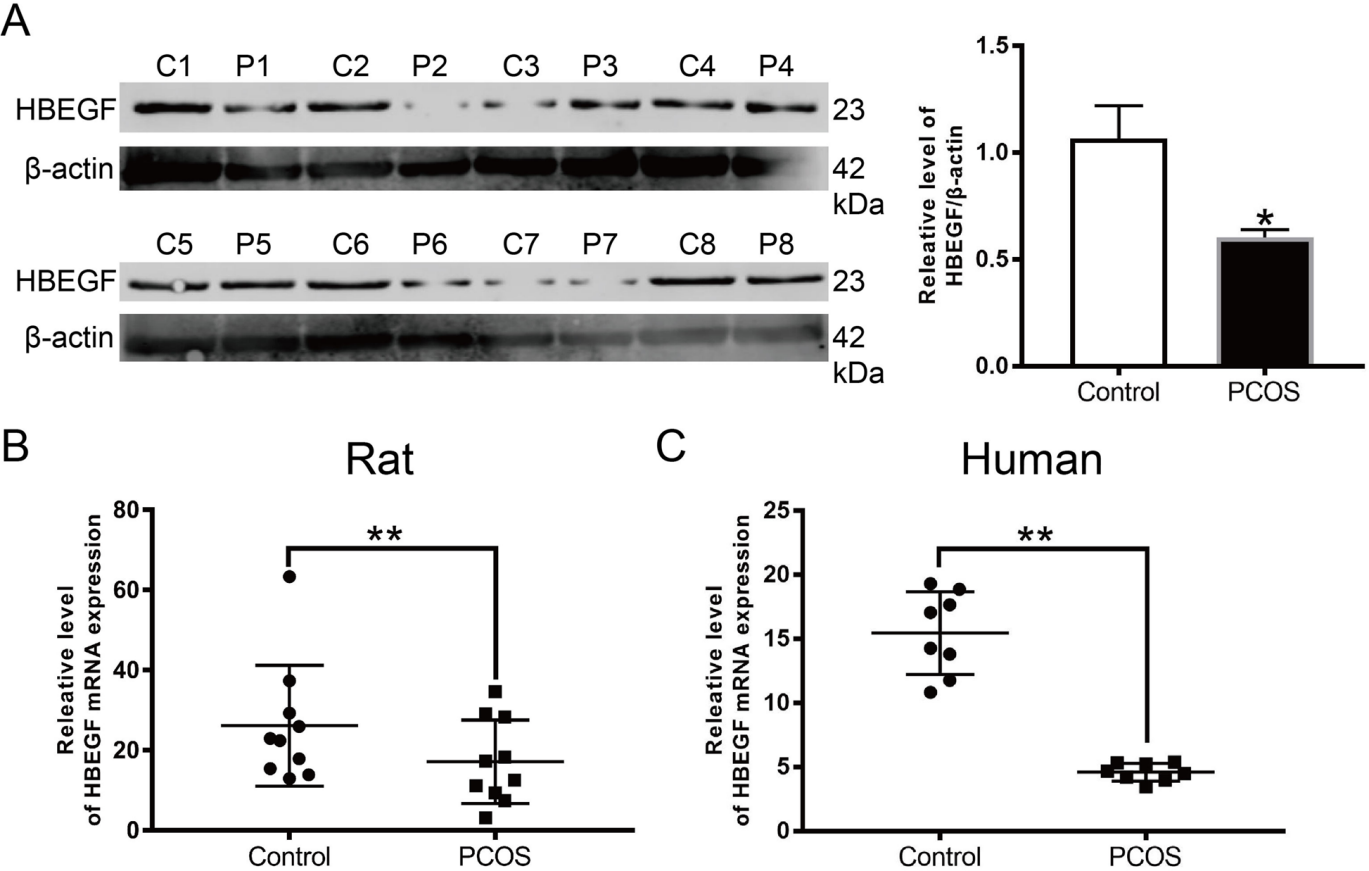
HB-EGF was decreased in ovarian tissues from PCOS rat model and PCOS GCs. (**A**) Western blotting and (**B**) qRT-PCR were used to quantify HB-EGF protein and mRNA levels in the PCOS rat model. (**C**) qRT-PCR demonstrate HB-EGF mRNA expression in PCOS patient GCs and the control group (n=10). B-C. 𝑛 = 3-6 for each group. *P < 0.05 vs. control group

To further confirm the role and effect of HB-EGF in PCOS, we first evaluated HB-EGF expression in ovarian tissues from a PCOS rat model (Fig. 4 A, B) and PCOS GCs (Fig. 4 C). HB-EGF was drceased in GCs from PCOS patients and the PCOS rat model in contrast to the normal group, based on qRT-PCR and Western blotting.

### HB-EGF upregulation reversed the impact of miR-194 mimic on KGN cell apoptosis

**Fig. 5:**
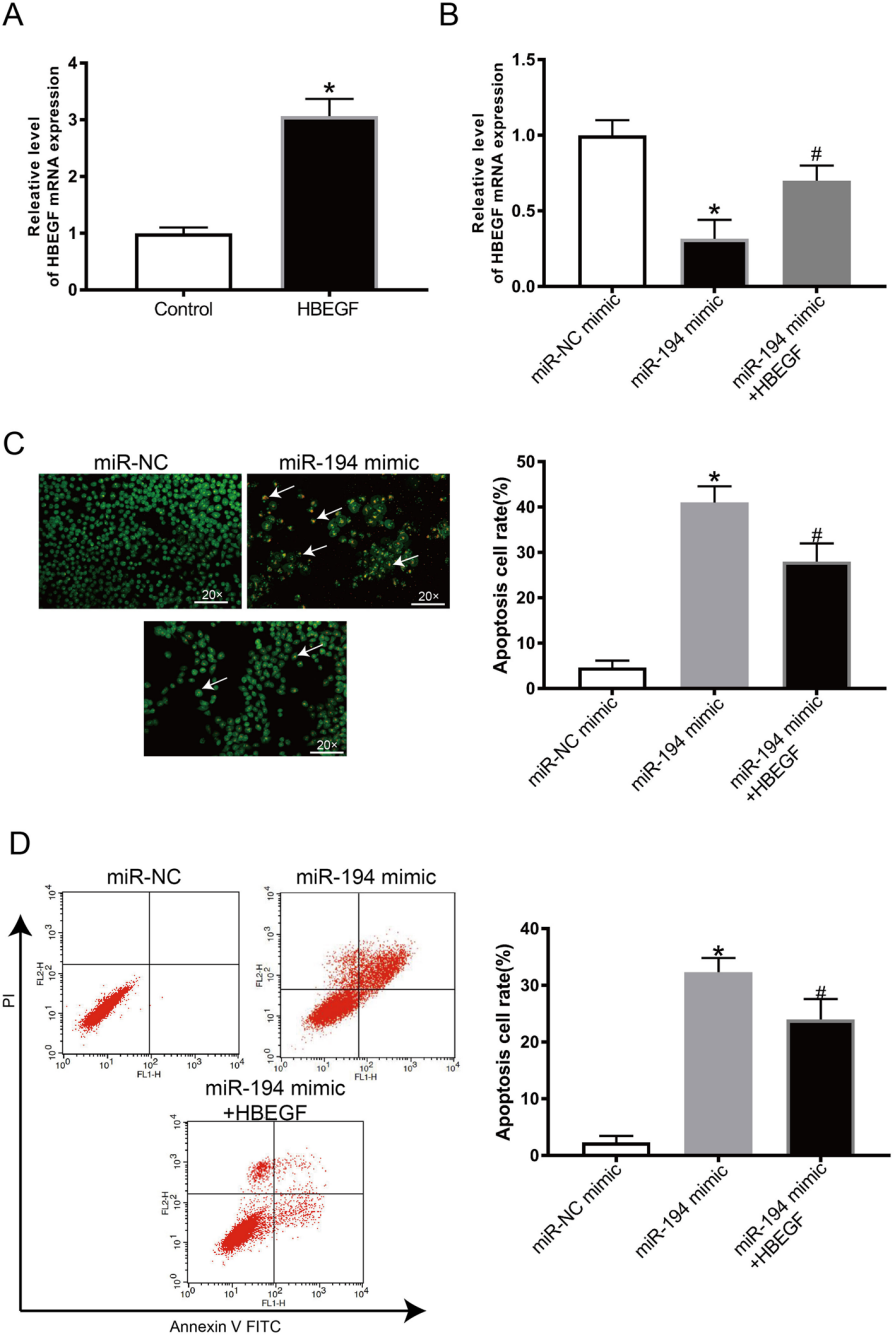
Upregulated HB-EGF reverses the effect of miR-194 mimic in KGN cells growth and apoptosis. (**A**) The expression of HB-EGF in KGN cells post-transfection with HB-EGF-OE. (**B**) The expression of HB-EGF in KGN cells post transfection with miR-194 mimic or miR-194 mimic+HB-EGF-OE. (**C**-**D**) The apoptotic rate of KGN cells was determined using AO/EB and flow cytometry. 𝑛 = 3-6 for each group; *P < 0.05 vs. miR-NC mimic group

We then explored the potential involvement of HB-EGF in the pathogenesis of PCOS. Rescue experiments were used to determine the relationship between miR-194 mimic and HB-EGF overexpression (HB-EGF-OE). We first used KGN cells transfected with either a miR-194 mimic or an HB-EGF-OE. As shown in Fig. 5 A, the expression of HB-EGF was up-regulated in KGN cells. In Fig. 5B, HB-EGF was remarkably downregulated when cells were transfected with a miR-194 mimic, while HB-EGF reversed this trend. AO/EB and flow cytometry were used to assess KGN cell apoptosis when co-transfected with miR-194 mimic or miR-194 mimic+ HB-EGF-OE (Fig. 5 C, D). HB-EGF upregulation rescued KGN cell apoptosis induced by miR-194 mimic alone.

## Discussion

PCOS is an endocrine-related ovarian defect with complex etiologic factors that adversely affect reproductive-aged women. The leading cause of infertility is a disorder in follicle development, which is stagnated in the antrum follicle stage (L J Webber et al. 2003, Franks S et al. 2008). Diabetes mellitus (DM) is characterized by hyperglycemia and hyperinsulinemia; PCOS patients exhibit the same symptoms [29–31]. Ovarian GCs form gap junctions with oocytes and play an essential role in the growth and development of oocytes [32, 33]. Thus, PCOS development has been established as a product of aberrant GC proliferation and apoptosis [34–36]. Notably, miRNAs have critical functions in a myriad of diseases, including PCOS [37]. Previous studies have shown that miRNA is abnormally expressed in follicular fluid and has been shown to modulate follicular development in human GCs. miR-194 has been implicated in several different malignancies, such as gastric cancer cell growth and lung cancer cell apoptosis [38, 39]. however, the role and effect of miR-194 in ovarian GCs are not entirely understood.

For the first time, the present study characterized miR-194 and HB-EGF expression in the GCs of PCOS patients and a PCOS rat model. Our results showed that miR-194 expression was up-regulated in PCOS GCs and a PCOS rat model, while there was an opposite effect on HB-EGF. We also demonstrated the relationship between miR-194 and HB-EGF, which shed light on the potential mechanism underlying GC growth and apoptosis. This series of experiments revealed that miR-194 induced GC apoptosis while likely inhibiting proliferation by targeting HB-EGF.

Evidence suggests that HB-EGF supports embryonal development, as well as the result of certain diseases [21]. Fontaine et al. reported that HB-EFG maintains myogenic tone in small vessel disease (Sethuraman et al., 2018). In contrast, HB-EGF protects against phenotypes related to metabolic syndrome and advanced metabolic disorders, suggesting that HB-EGF is a potential target against metabolic diseases, such as hyperinsulinemia, hyperglycemia, and increased oxidative stress, all of which are in common with insulin dysregulation [18]. Our study provides the first genetic evidence that HB-EGF plays a critical role in PCOS progression. In the present study, HB-EGF mRNA and protein expression were decreased in PCOS GCs and a PCOS rat model. Herein we demonstrated elevated HB-EGF expression in PCOS and up-regulated HB-EGF expression rescue increased the GC apoptosis rate triggered by exposure of the cells to a miR-194 mimic. This finding is consistent with HB-EGF amelioration of oxidative stress-mediated uterine decidualization damage, downregulation of circulatory lipid levels, protection against atherosclerosis in the vascular wall, and attenuation of lung inflammation and injury in a murine model of pulmonary emphysema (Hai-Fan Yu et al. 2019,S Kim et al. 2019,Yanwei Su et al. 2019). .

It has been reported that miR-194 regulates various diseases by targeting genes, such as GRB2-associated binding protein 1 (GAB1) [40], nuclear receptor subfamily 2 group F member 2 (NR2F2) [41], NLRP3 [42], and SLC40A1 [43]. miRNA regulates post-transcription involved in PCOS-related molecular expression [44, 45]. We explored the potential biological functions of miR-194 in PCOS development by first predicting potential gene targets of miR-194 using bioinformatics tools (Fig. 3). HB-EGF stands out from all other potential target genes given its involvement in many other cancers, such as gliomagenesis [46], breast cancer [47], and medullary thyroid carcinoma [48]. In addition, previous studies also showed that HB-EGF mediates proliferation and apoptosis [49]. Notably, HB-EGF is a direct target gene of miR-183, which has been shown to impede embryo implantation in the mouse uterus [50]. These studies suggest a close association between HB-EGF and abnormal cell growth. At the same time, we test the p53 signaling pathway rela-proteins. Our results showed over-expression of miR-194 can up-regulate the protein level of p53, p21, and p16. Similarly, Zhang et al. suggest that the p53 signaling pathway plays an essential role in the apoptosis of GCs [51]. Liu C et al. reported that p53 deacetylation is a crucial step of miR-874-3p induce GCs apoptosis [12]. miR-3188 promoted the cell cycle by decreasing p21 in KGN cells [52]. And also, cellular stresses are activated in response to p53 signaling through HB-EGF induction [53]. These findings indicate that the p53 signaling pathway is an important role in miR-194/HB-EGF regulation KGN cell apoptosis.

The main limitations of our study were as follows: (i) the small GC samples of clinical patients; (ii) GCs obtained from IVF follicular may contain other impure cells (Adams et al., 2021). In the current study, miR-194 and HB-EGF expression in PCOS GCs was consistent with the PCOS rat model. It is a notable finding and warrants further research to understand the pathophysiologic mechanism underlying PCOS better.

In conclusion, our data uncovered the role of miR-194 in KGN cells growth and apoptosis. miR‐194 overexpression inhibited KGN cells growth and induced apoptosis via direct HB-EGF targeting. Taken together, miR‐194/HB-EGF may have the potential to function as a novel biomarker and therapeutic target for PCOS.

## Supplementary Information

**Additional file 1.**

**Additional file 2.**

## Data Availability

The datasets used and/or analyzed during the current study are available from the corresponding author on reasonable request.
