# Supplementary figures and images for: microRNA-194 is increased in polycystic ovary syndrome granulosa cell and induce KGN cells apoptosis by direct targeting heparin-binding EGF-like growth factor
Source: Reprod Biol Endocrinol. 2021 Nov 23;19:170. doi: 10.1186/s12958-021-00850-w (PMC8609843; doi:10.1186/s12958-021-00850-w)

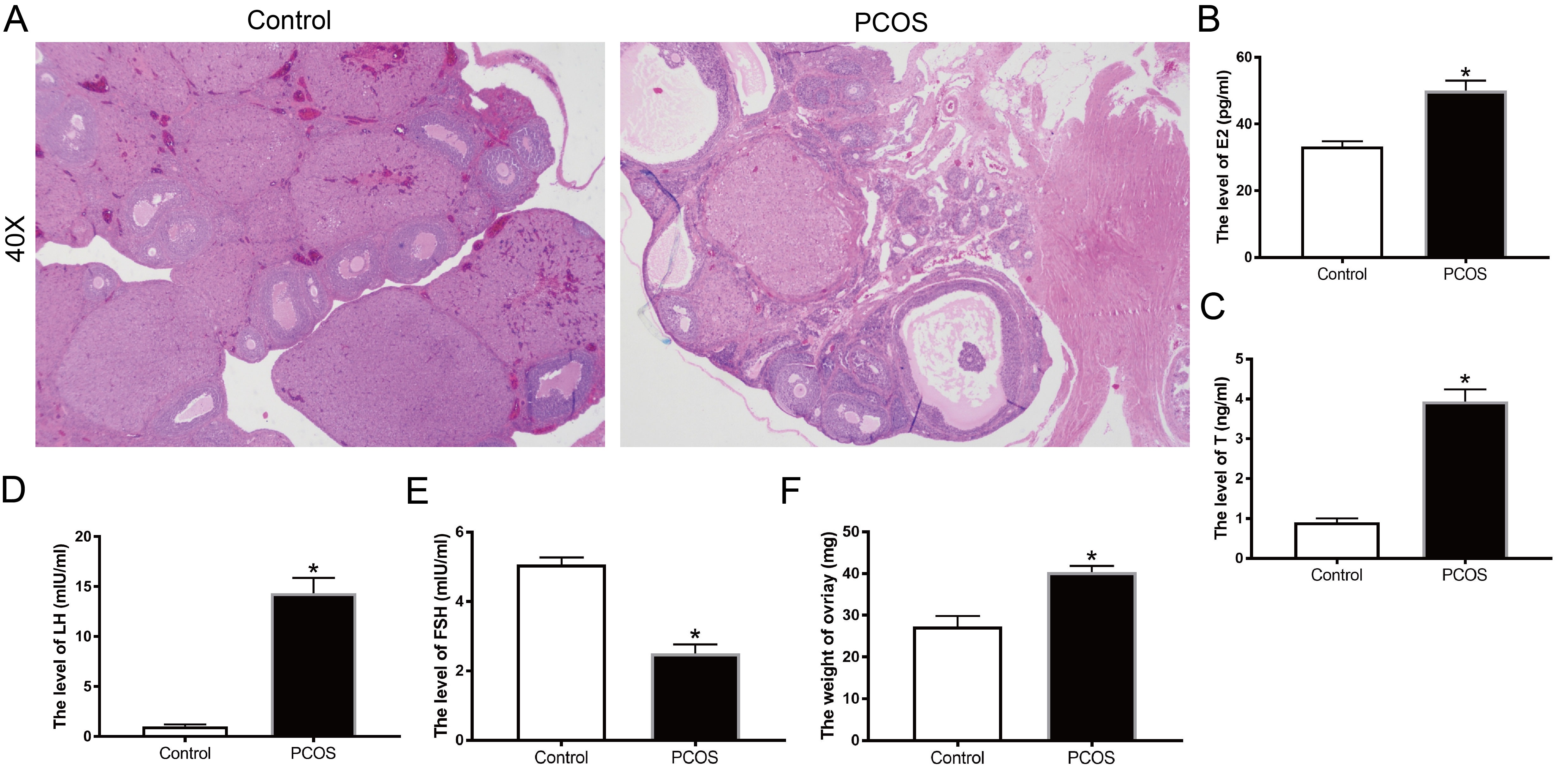

Supplement: Supplementary file 1 — Additional file 1. [file 12958_2021_850_MOESM1_ESM.jpg]

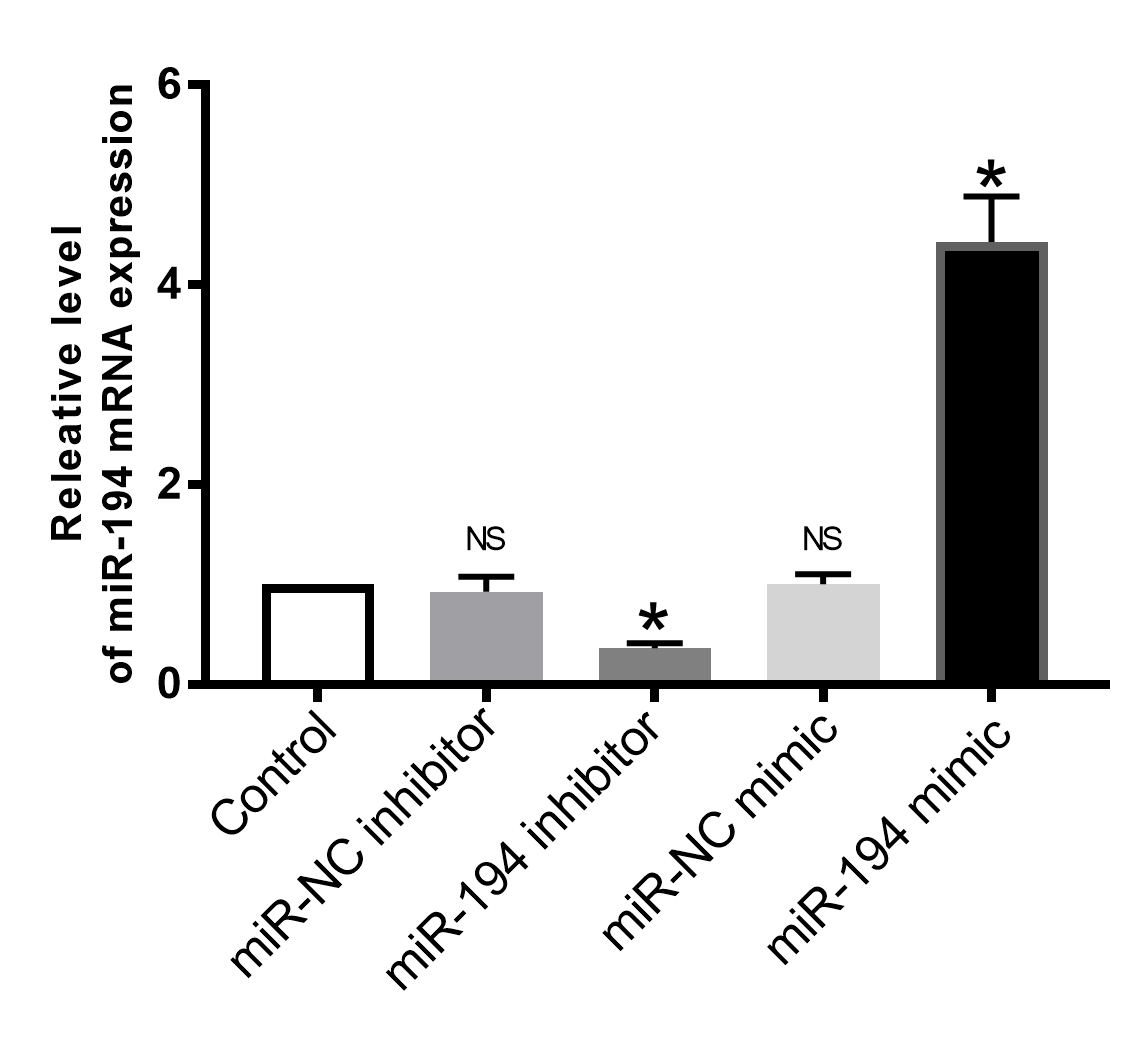

Supplement: Supplementary file 2 — Additional file 2. [file 12958_2021_850_MOESM2_ESM.tif]
